# Vagus nerve stimulation as a predictive coding modulator that enhances feedforward over feedback transmission

**DOI:** 10.3389/fncir.2025.1568655

**Published:** 2025-04-14

**Authors:** Shinichi Kumagai, Tomoyo Isoguchi Shiramatsu, Kensuke Kawai, Hirokazu Takahashi

**Affiliations:** ^1^Department of Neurosurgery, Jichi Medical University, Tochigi, Japan; ^2^Department of Mechano-Informatics, Graduate School of Information Science and Technology, The University of Tokyo, Tokyo, Japan

**Keywords:** vagus nerve stimulation, acetylcholine, noradrenaline, plasticity, cognitive function, epilepsy, depression, stroke rehabilitation

## Abstract

Vagus nerve stimulation (VNS) has emerged as a promising therapeutic intervention across various neurological and psychiatric conditions, including epilepsy, depression, and stroke rehabilitation; however, its mechanisms of action on neural circuits remain incompletely understood. Here, we present a novel theoretical framework based on predictive coding that conceptualizes VNS effects through differential modulation of feedforward and feedback neural circuits. Based on recent evidence, we propose that VNS shifts the balance between feedforward and feedback processing through multiple neuromodulatory systems, resulting in enhanced feedforward signal transmission. This framework integrates anatomical pathways, receptor distributions, and physiological responses to explain the influence of the VNS on neural dynamics across different spatial and temporal scales. Vagus nerve stimulation may facilitate neural plasticity and adaptive behavior through acetylcholine and noradrenaline (norepinephrine), which differentially modulate feedforward and feedback signaling. This mechanistic understanding serves as a basis for interpreting the cognitive and therapeutic outcomes across different clinical conditions. Our perspective provides a unified theoretical framework for understanding circuit-specific VNS effects and suggests new directions for investigating their therapeutic mechanisms.

## Introduction

Vagus nerve stimulation (VNS) has emerged as a promising therapeutic approach for various neurological and psychiatric disorders. This neuromodulatory technique, which involves electrical stimulation of the vagus nerve, has gained significant attention due to its broad therapeutic potential. The U.S. Food and Drug Administration (FDA) has approved VNS for several therapeutic applications. Its primary indication is treatment of drug-resistant epilepsy (Afra et al., 2021). The efficacy of VNS in reducing seizure frequency and severity has been demonstrated in numerous clinical trials in patients with intractable epilepsy (George et al., 1995; Handforth et al., 1998; Englot et al., 2011). In addition to epilepsy, VNS has also received FDA approval for the management of treatment-resistant depression. Studies have shown promising results in alleviating depressive symptoms in patients who do not respond adequately to conventional therapies including medication and psychotherapy (Rush et al., 2005; Schlaepfer et al., 2008; Aaronson et al., 2013; Aaronson et al., 2017). Non-invasive VNS has been approved for the acute treatment of migraine, and clinical trials have demonstrated pain relief after attacks with stimulation (Tassorelli et al., 2018; Martelletti et al., 2018). More recently, VNS has been approved as an adjunct therapy in stroke rehabilitation. Vagus nerve stimulation, when paired with motor rehabilitation, can enhance motor function recovery in stroke survivors (Dawson et al., 2016; Dawson et al., 2021; Kwakkel and Dobkin, 2021). These clinical effects are likely mediated through the modulation of various neurotransmitters including acetylcholine, noradrenaline, and serotonin (Krahl et al., 1998; Raedt et al., 2011; Fornai et al., 2011; Furmaga et al., 2011; Zhang et al., 2019; Hulsey et al., 2016; Engineer et al., 2019; Hulsey et al., 2019; Collins et al., 2021; Carron et al., 2023).

The potential benefits of VNS extend beyond the approved indications. Numerous studies have suggested that VNS may positively affect cognitive function. Studies have explored its impact on various aspects of cognition, including memory, executive function, and attention, in patients with epilepsy and mild cognitive impairment, and in healthy adults (Mertens et al., 2022; Aniwattanapong et al., 2022; Wang et al., 2022; Klaming et al., 2022). However, it is crucial to acknowledge that the effects of VNS on cognitive processes are complex and not uniformly positive. While many studies have reported improvements, some have found no significant changes or even potential impairments in certain cognitive domains following VNS treatment (Clark et al., 1999; Helmstaedter et al., 2001; Ghacibeh et al., 2006; McGlone et al., 2008; Vonck et al., 2014; Mertens et al., 2020; Kong et al., 2024). These varied outcomes underscore the complex interactions between VNS and cognitive processes.

This perspective aims to explore the intricate relationship between VNS and brain information processing. We propose a novel framework to elucidate the mechanisms underlying the diverse effects of VNS on the neural circuitry.

## A novel framework for circuit-specific VNS effects

**Figure 1:**
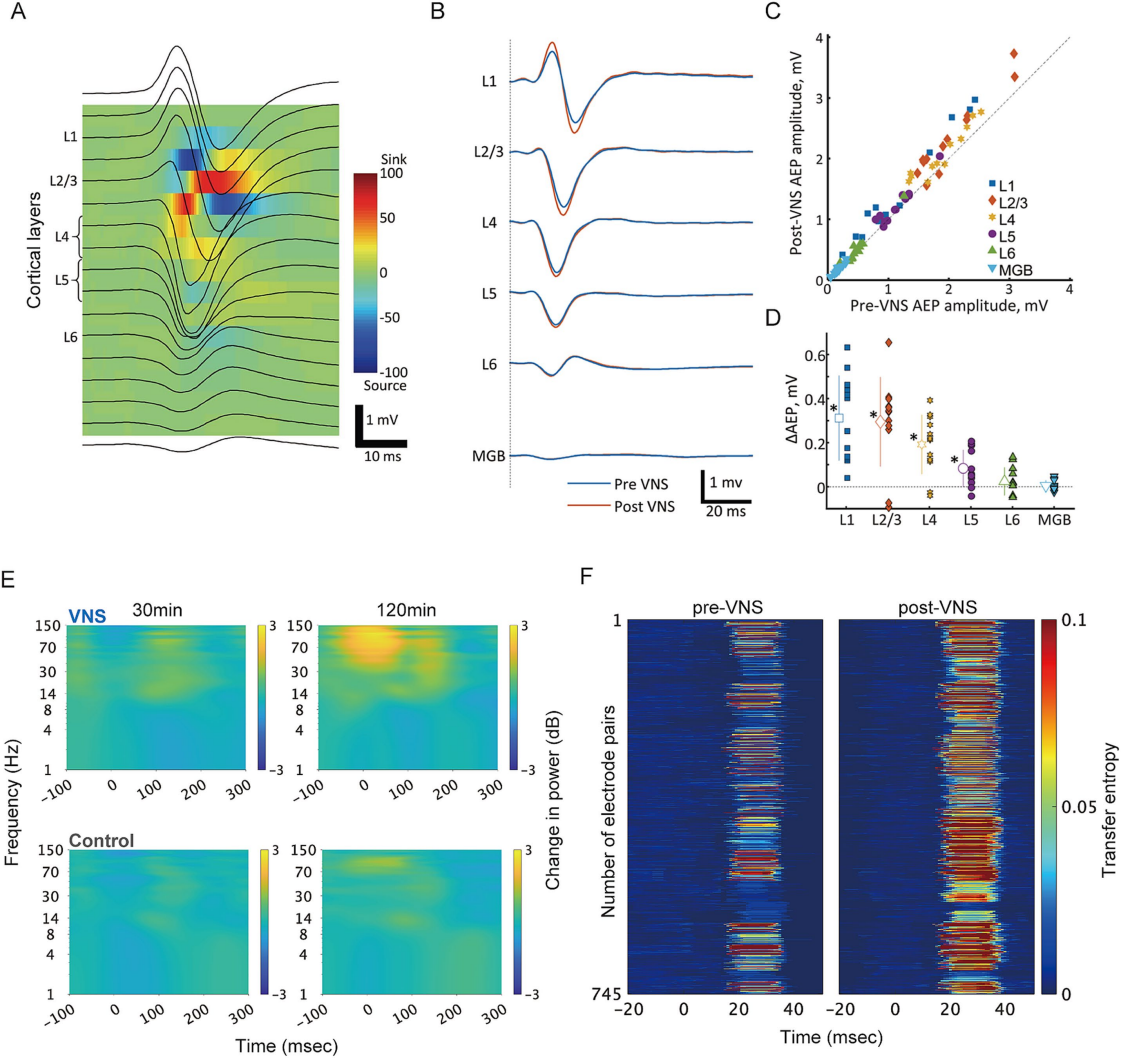
VNS differentially modulates cortical processing across layers and pathways in the auditory system. **(A)** Current source density (CSD) analysis for layer identification in the rat auditory cortex. Representative laminar recordings showing auditory evoked potentials (AEPs, black traces) superimposed on the color-coded CSD map. The characteristic pattern of current sinks (red) and sources (blue) enabled precise identification of cortical layers L1-L6. Scale bar: 1 mV, 10 ms. **(B)** VNS-induced modulation of click-evoked responses across cortical layers. Grand-averaged AEP waveforms recorded simultaneously from different cortical layers before (blue) and after (red) VNS. Layer-specific enhancement was observed in response amplitudes. **(C)** Quantitative comparison of AEP amplitudes between pre- and post-VNS conditions. Scatter plot of AEP amplitudes showing individual recording sites across different layers, with each point representing pre- vs. post-VNS amplitudes. Points above the diagonal line indicate VNS-induced enhancement. **(D)** Layer-specific profile of VNS effects. The magnitude of VNS-induced changes in AEP amplitude (ΔAEP) showed layer-dependent differences. Comparable enhancement was observed in superficial layers, whereas this effect diminished in deeper layers. Data points represent individual recordings; bars indicate mean ± SD. Asterisks denote statistical significance (*p < 0.05). **(E)** Time-frequency analysis of VNS-induced modulation of oscillatory activity in response to click sounds. Spectrograms comparing VNS (top) and control (bottom) groups showing the temporal evolution of frequency-specific power in the rat auditory core region at early (30 min) and late (120 min) timepoints during click presentation experiments. The color scale (right) indicates power changes in decibels relative to baseline period (−500 to −200 msec). The VNS group demonstrated progressive enhancement of high-frequency oscillations (gamma: 30–150 Hz) accompanied by attenuation of low-frequency activity (theta: 4–8 Hz). In contrast, the control group showed minimal changes in oscillatory patterns over time. Data were averaged across recording sites within the functionally defined auditory core region. **(F)** Information flow analysis reveals VNS-induced modulation of feedforward pathways in the rat auditory cortex. Transfer entropy (TE) analysis was performed on multi-unit recordings from functionally connected electrode pairs in the core and belt regions during click-evoked responses. Each row represents individual electrode pairs from a single rat. Left: Color-coded normalized TE values for feedforward pathways through layer 4 before VNS. Right: Corresponding TE values after VNS, demonstrating enhanced information flow. TE analysis was performed following the methods described in Ishizu et al. (2021). CSD, current source density; AEP, auditory evoked potential; VNS, vagus nerve stimulation; MGB, medial geniculate body in the ventral division of thalamus; L, layer; TE, transfer entropy.

Although numerous studies have demonstrated the efficacy of VNS in various neurological and psychiatric conditions, the underlying neural processes are not fully understood. Our recent studies provided initial insights into the layer-specific effects of VNS on sensory processing. We demonstrated that VNS predominantly enhanced auditory-evoked responses in the superficial layers of the primary auditory cortex (A1), with diminishing effects in the deeper layers (Takahashi et al., 2020) (Figures 1A–D). Furthermore, we found that VNS modulates oscillatory activities in A1 through the cholinergic and noradrenergic systems, suggesting distinct modulation of cortical oscillations (Kumagai et al., 2023) (Figure 1E). Additionally, our preliminary data showing changes in functional connectivity between the core and belt regions in the auditory cortex provides further evidence for the pathway-specific effects of VNS (Figure 1F). Specifically, VNS enhanced the transfer entropy of evoked activity from the core to the belt regions, suggesting a strengthening of feedforward information flow in the auditory cortex.

Based on these findings and several lines of supporting evidence, we propose a novel hypothesis: VNS modulates the balance between feedforward and feedback processing, specifically enhancing feedforward information flow in the thalamo-cortical and cortico-cortical systems. This modulation can be interpreted within the framework of predictive coding, which proposes that the brain continuously generates predictions about incoming sensory inputs and minimizes the difference (prediction error) between these predictions and the actual inputs (Heilbron and Chait, 2018). Predictive coding has been mathematically formalized under the free energy principle, which quantifies the discrepancy between the brain’s internal models and actual sensory inputs using variational Bayesian inference. According to this theory, prediction errors propagate from lower to higher areas in feedforward pathways, whereas predictions propagate in the opposite direction in feedback pathways (Friston and Kiebel, 2009; Bastos et al., 2012; Aitchison and Lengyel, 2017). In the auditory system, each level of processing compares incoming acoustic signals with what the brain expects to hear, generating prediction errors when there are mismatches between expected and actual sounds. These prediction errors flow upward from the primary auditory cortex through the higher auditory regions, whereas expectations about upcoming sounds flow downward. For example, when we listen to speech and hear an unexpected pronunciation or an unfamiliar accent, the brain initially generates large prediction errors; as we continue to listen, these prediction errors trigger an update of our predictions about the speaker’s speech patterns, gradually reducing subsequent prediction errors and improving our ability to understand the speaker’s speech.

In this predictive coding framework, VNS may enhance the feedforward signaling of prediction errors with respect to feedback signaling of prediction, thereby influencing how effectively the brain processes sensory information and updates its internal models. Multiple lines of evidence support this hypothesis at anatomical, physiological, and functional levels.

## Anatomical basis for circuit-specific modulation

**Figure 2:**
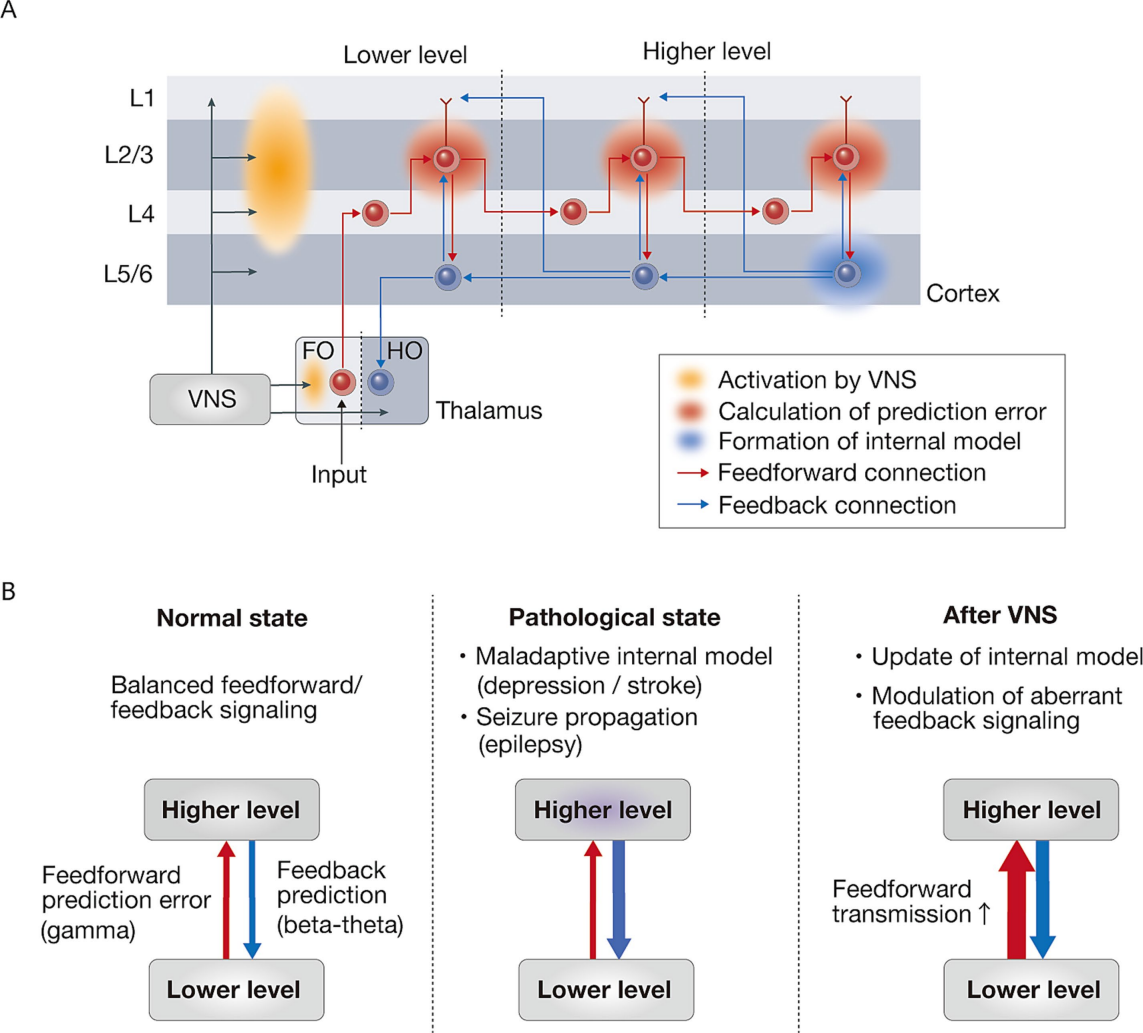
VNS-induced modulation of neural information processing through feedforward-feedback interactions. **(A)** Cortical laminar organization of feedforward and feedback signals in prediction error processing. Prediction errors are computed in layer 2/3 (supragranular layers) through the integration of multiple inputs: feedback predictions from higher cortical areas arriving via layer 1, local predictions from layer 5/6 (infragranular layers), and feedforward sensory inputs from lower cortical areas. This hierarchical process allows layer 2/3 neurons to compare incoming sensory signals against predictions, enabling the computation of prediction errors through complex multi-layered processing. These computed prediction errors are then propagated in a feedforward manner, thereby continuously updating internal models in higher cortical areas, enabling adaptive predictions about incoming sensory information. VNS may predominantly enhance the activation of superficial layers compared to deep layers, potentially facilitating the feedforward transmission of prediction errors throughout the cortical hierarchy. The thalamic components are represented as FO (first-order) nuclei, which receive ascending sensory inputs, and HO (higher-order) nuclei, which primarily receive cortical inputs. **(B)** Schematic illustration of hierarchical information processing in normal, pathological, and VNS-treated states. In the normal state, there is a balanced interaction between feedforward prediction error (gamma) and feedback prediction (beta-theta) signaling. The pathological state is characterized by maladaptive internal model (depression / stroke) and seizure propagation (epilepsy). VNS therapy enhances feedforward transmission, which leads to updating of the internal model and modulation of aberrant feedback signaling. Red and blue arrows indicate feedforward and feedback signaling, respectively. VNS, vagus nerve stimulation.

To understand how VNS might differentially affect feedforward and feedback processing, it is essential to consider the underlying anatomical organization of the cortical circuits. The cerebral cortex exhibits a hierarchical organization defined by distinct laminar patterns of inter-areal connections that support predictive coding processes (Figure 2A). In the cortical hierarchy, feedforward projections originate primarily from the supragranular layers (layers 2/3) and target layer 4, while feedback projections arise from the infragranular layers (layers 5/6) and terminate in layers 1 and 6 (Bastos et al., 2012; Felleman and Van Essen, 1991; Shipp, 2007). Although detailed anatomical studies have shown that both supragranular and infragranular layers contain feedforward and feedback streams, feedforward and feedback pathways predominantly use the supragranular and infragranular layers, respectively (Markov et al., 2014).

The supragranular layers exhibit precise point-to-point connectivity, enabling accurate signal transmission between specific cortical regions, which is crucial for feedforward sensory processing (Markov et al., 2013). In contrast, the infragranular layers show more diffuse connectivity patterns, facilitating broad signal integration and divergence across multiple areas, which is particularly important for feedback signaling (Markov et al., 2014). This dual organization allows the cortex to simultaneously achieve both precise and rapid information processing through targeted feedforward pathways, and flexible contextual interpretation through distributed feedback networks. Such anatomical organization provides the structural basis for predictive coding, where prediction errors are primarily conveyed through supragranular layers from lower to higher areas, whereas predictions are transmitted through infragranular layers from higher to lower areas (Friston and Kiebel, 2009).

Vagus nerve stimulation predominantly enhances neural responses in superficial layers (I-IV), with diminishing effects in deeper layers (Takahashi et al., 2020). This laminar specificity of VNS effects aligns with the anatomical organization of feedforward pathways, which primarily originate from superficial layers. Since these layers are the main source of projections to higher cortical areas, VNS-induced enhancement of activity in superficial layers selectively strengthens ascending sensory information flow through the cortical hierarchy. These findings provide mechanistic evidence that VNS selectively enhances feedforward information flow in the cortical circuits.

## Receptor distribution of neuromodulatory transmitters

The proposed mechanism of VNS-induced enhancement of feedforward processing can be further supported by examining the detailed anatomical laminar organization of the neuromodulatory systems. The distribution of cholinergic and noradrenergic receptors shows distinct layer-specific patterns, which aligns with this hypothesis.

The cholinergic inputs show a denser distribution in superficial layers (I-IV) associated with feedforward processing, with both nicotinic and muscarinic receptors enriched in these layers (Palomero-Gallagher and Zilles, 2019). This organization suggests a potential mechanism by which VNS selectively modulates feedforward pathways.

The adrenergic receptor distribution exhibits intricate layer-specific patterns that may contribute to the proposed feedforward enhancement. α1- and α2-adrenergic receptors tend to be densely distributed in the superficial layers across many cortical regions, although their specific distribution patterns vary between different cortical areas (Palomero-Gallagher and Zilles, 2019). β-adrenergic receptors are predominantly localized in the deep cortical layers (V and VI) of the sensory cortex of young cats (Liu et al., 1993). During development, this distribution pattern shifts, ultimately establishing an adult pattern with two distinct bands of high receptor density in superficial and deep cortical layers.

The varying affinities of different adrenergic receptor subtypes (*α*2 highest, followed by α1, and then β) suggest that VNS may produce concentration-dependent effects. Indeed, noradrenaline exhibits dual effects on glutamate-evoked neuronal discharge through distinct receptor subtypes: α1-receptor activation results in facilitation, whereas β-receptor activation leads to the suppression of these glutamate-evoked responses (Devilbiss and Waterhouse, 2000). Moderate noradrenaline concentrations may preferentially activate α1-receptors in superficial layers, enhancing feedforward signaling, whereas higher concentrations could additionally recruit β-receptors in deep layers, potentially suppressing the influence of feedback pathways. Consistent with the suppressive role of the β-receptor, a pharmacological study has shown that β-receptor activation impairs prefrontal cortical cognitive function in both rats and monkeys (Ramos et al., 2005).

This differential activation of the adrenergic receptor subtypes affects VNS-induced plasticity. α- and β-adrenergic receptors can exert opposing effects on synaptic plasticity in a noradrenaline concentration-dependent manner (Salgado et al., 2012), which may explain why VNS-induced cortical plasticity exhibits an inverted-U relationship with stimulation intensity (Hays et al., 2023). At moderate intensities, plasticity is optimized, whereas both low and high intensities are less effective (Morrison et al., 2019). α2-receptor activation in the motor cortex is necessary for VNS-induced plasticity at a moderate (0.8 mA) stimulation intensity (Tseng et al., 2021).

Collectively, these findings suggest that VNS modulates the balance between feedforward and feedback circuits through intensity-dependent and receptor-specific mechanisms that are mediated by the differential anatomical distribution of receptor subtypes. This receptor-based mechanism provides a neurochemical foundation for how VNS enhances feedforward information flow while potentially attenuating feedback influences, supporting our proposed theoretical framework.

## VNS-induced modulation of neural oscillations

Supporting our hypothesis that VNS differentially modulates feedforward and feedback processing, electrophysiological evidence has demonstrated specific alterations in neural oscillations and information transmission patterns. Neural oscillations exhibit distinct characteristics across different spatial scales and laminar distributions. Gamma oscillations tend to coordinate local circuit activities such as sensory processing and are predominantly generated in the superficial cortical layers. In contrast, slower oscillations, such as theta waves, are more prominent in the deeper layers and facilitate communication between distant brain regions (Bastos et al., 2012; Sarnthein et al., 1998; Miller et al., 2018). These oscillations can interact hierarchically through cross-frequency coupling, providing a mechanism for integrating bottom-up sensory information with top-down cognitive control (Fries, 2023).

Vagus nerve stimulation enhances auditory-evoked gamma power through the cholinergic system and decreases theta power through the noradrenergic system in the rat auditory cortex (Kumagai et al., 2023). Furthermore, our recent decoding analysis shows that stimulus information represented by gamma-band activity increases in the superficial layer and decreases in the deep layer during VNS (Shiramatsu et al., 2025). These data suggest that VNS strengthens feedforward pathways through the cholinergic system, while attenuating feedback circuits via the noradrenergic system. The VNS-induced enhancement of gamma oscillations indicates increased feedforward signaling, which is consistent with established evidence that gamma band activity mediates feedforward information flow (Bastos et al., 2015; Chao et al., 2018; Bastos et al., 2020; Vezoli et al., 2021). However, the functional significance of VNS-induced theta suppression requires further investigation. Although theta rhythms may contribute to both feedforward and feedback processing (Bastos et al., 2015; Chao et al., 2018; Bastos et al., 2020; Vezoli et al., 2021), they are commonly linked to top-down control (Sarnthein et al., 1998; Cavanagh and Frank, 2014), which involves the coordination of distributed neural networks to implement cognitive functions such as working memory (Sarnthein et al., 1998). In this context, VNS-induced enhancement of gamma oscillations coupled with theta suppression may represent a shift in cortical processing that favors feedforward sensory transmission while reducing top-down feedback influences, potentially optimizing the balance between bottom-up and top-down information flow.

Further evidence comes from an EEG study of non-invasive VNS in healthy subjects, which decreased theta and alpha power while increasing beta and gamma power, indicating a shift toward cortical activation (Lewine et al., 2019). Consistent with these findings, intraoperative electrocorticographic recordings in patients with refractory epilepsy demonstrated that VNS significantly enhanced high-frequency spectral power, particularly in the beta and gamma bands (Yokoyama et al., 2020). A recent multicenter study examining intracranial recordings in patients with epilepsy who had both VNS and responsive neurostimulation (RNS) systems demonstrated significant reductions in the theta-band power during VNS (Ernst et al., 2023). Similarly, transcutaneous VNS leads to pupil dilation and attenuation of occipital alpha oscillations, suggesting increased arousal and potentially enhanced sensory processing (Sharon et al., 2021). However, a replication study observed increased pupil size but failed to replicate the alpha attenuation (Lloyd et al., 2023), and an early study of invasive VNS in epilepsy patients found no significant effects on awake EEG background rhythms (Salinsky and Burchiel, 1993). Furthermore, both invasive and non-invasive VNS modulate low-frequency spectral power across distributed cortical networks, but the effects vary considerably based on stimulation parameters and individual differences (Schuerman et al., 2021).

While these mixed findings of oscillatory activity suggest variable effects on resting-state brain activity, more consistent effects emerge when examining active sensory processing, likely due to VNS modulation of engaged feedforward and feedback circuits in response to sensory stimuli. Information theoretical analyses have demonstrated that VNS rapidly enhances the feature selectivity and information transmission of thalamic neurons in the rat somatosensory system (Rodenkirch and Wang, 2020). This improvement coincided with the suppression of the thalamic burst firing. Given that the thalamus plays a crucial role in relaying sensory information to the cortex, these findings suggest that VNS specifically enhances feedforward sensory processing through more precise and efficient feedforward signaling from the thalamus to the primary sensory cortex.

Together, these electrophysiological findings strongly support our hypothesis that VNS differentially modulates feedforward and feedback neural transmission, primarily through enhancement of feedforward pathways. This modulation appears to occur through rapid activation of distinct neuromodulatory systems, potentially explaining the observed effects on sensory processing.

## Neuromodulatory control of neural plasticity

The understanding of neuromodulatory control over cortical plasticity has evolved significantly over the past three decades. Early investigations in the 1990s demonstrated that sensory experience alone was insufficient to drive cortical plasticity and revealed that both sensory experience and neuromodulatory signaling are essential to induce long-lasting changes in cortical circuits (Bakin and Weinberger, 1996; Kilgard and Merzenich, 1998a; Kilgard and Merzenich, 1998b). These pioneering insights into neuromodulatory mechanisms formed the basis for subsequent investigations into VNS-induced plasticity. Our hypothesis of VNS-enhanced feedforward processing also builds upon these foundational studies of neuromodulatory control over cortical plasticity: enhancement of the feedforward pathway, i.e., prediction error, would promote plasticity and update generative models in higher-order cortical areas.

Early studies have demonstrated that the cholinergic system plays a crucial role in cortical plasticity, finding that pairing auditory stimuli with nucleus basalis stimulation induces long-lasting changes in auditory cortical receptive fields (Bakin and Weinberger, 1996). This form of plasticity shared key features with behavioral memory: it was associative, specific, and long-lasting (McLin et al., 2002; Weinberger, 2003; Weinberger, 2004; Weinberger, 2007). Nucleus basalis stimulation paired with tones induces large-scale reorganization of frequency representation in the auditory cortex (Kilgard and Merzenich, 1998a; Kilgard and Merzenich, 1998b), triggering a sequence of synaptic changes: rapid disinhibition followed by delayed enhancement of excitation (Froemke et al., 2007). Paring sensory stimuli with nucleus basalis stimulation enhances sensory processing in two ways: it improves the reliability of neuronal responses and reduces correlations between cortical neurons, resulting in improved perceptual performance in behaving animals (Goard and Dan, 2009; Froemke et al., 2013). Similarly, VNS paired with tones induced targeted plasticity in the auditory cortex and eliminated behavioral correlates of tinnitus in noise-exposed rats (Engineer et al., 2017; Engineer et al., 2011).

Like cholinergic modulation, noradrenergic signaling from the locus coeruleus (LC) powerfully influences cortical plasticity through distinct mechanisms. The activation of LC neurons can trigger long-lasting changes in auditory cortical responses (Martins and Froemke, 2015). Specifically, pairing tones with LC stimulation induced coordinated plasticity in both the modulatory and sensory pathways, leading to improved auditory perception that could last for weeks. Pairing auditory stimuli with LC stimulation induced two distinct patterns of plasticity in auditory cortical neurons: frequency-selective changes with an increase or decrease in evoked responses in more than 30% of neurons, and non-selective response changes across frequencies in approximately 50% of neurons (Edeline et al., 2011). This suggests that LC activation can simultaneously drive both stimulus-specific refinement and broader changes in the cortical excitability. In a more recent study, LC activation was found to facilitate cochlear implant-driven plasticity, significantly enhancing long-term perceptual performance in a rat model of cochlear implantation (Glennon et al., 2023). Initially, LC activation induced a widespread increase in auditory cortical responses, enhancing the activity to both paired and non-rewarded inputs. Over the course of training, these changes became progressively more selective, with LC activity increasingly favoring paired stimuli while suppressing responses to non-rewarded inputs.

While cholinergic modulation suppresses stimulus-evoked inhibition followed by selective enhancement of excitation to paired stimuli (Froemke et al., 2007; Froemke et al., 2013), noradrenergic modulation enhances the overall gain of cortical responses, showing increased activity to both paired and unpaired stimuli, with some preference for the paired frequency (Martins and Froemke, 2015). These distinct modulatory effects suggest different roles in cortical plasticity: selective modification of specific inputs by acetylcholine versus broader changes in cortical processing by noradrenaline (Martins and Froemke, 2015; Froemke, 2015). These studies on cholinergic and noradrenergic modulation help explain how VNS promotes plasticity through the engagement of these neuromodulatory systems (Hulsey et al., 2016; Hulsey et al., 2019; Hays et al., 2023; Tseng et al., 2021; Brougher et al., 2021; Bowles et al., 2022; Martin et al., 2024). These effects likely occur through vagal activation, first by engaging noradrenergic pathways, which then modulate cholinergic function, rather than through direct vagal-cholinergic projections (Collins et al., 2021).

These plasticity effects induced by VNS (or cholinergic activation) are dependent on the timing of the paired movement or stimulus (Bowles et al., 2022; Martin et al., 2024). As synaptic plasticity, i.e., potentiation or depotentiation in response to incoming input, should be effectively driven by prediction errors (Saponati and Vinck, 2023), the timing-dependent nature of the VNS effects suggests that cholinergic modulation emphasizes prediction error signals. The effectiveness of plasticity also depends on spatial and temporal precision and salience relative to the background neural activity, specifically during behaviorally relevant moments. Cholinergic signaling is likely to modulate normalization, through which neuronal activity is divided by the pooled activity of surrounding neurons, which is associated with reductions in noise correlations, minimization of trial-to-trial variability, and enhancement of the signal-to-noise ratio (Carandini and Heeger, 2011; Schmitz and Duncan, 2018). Through these normalization effects, cholinergic modulation potentially refines the neural representation of prediction error signals, allowing neural circuits to update their predictive models more efficiently, based on experience. Within our proposed framework, VNS strengthens this process by selectively enhancing feedforward connections that carry prediction error signals while simultaneously attenuating feedback influences, increasing the learning efficiency of neural systems. This shifted balance toward feedforward processing may explain why VNS paired with specific stimuli or movements induces more robust and enduring plasticity than experience alone.

## Dual neuromodulatory systems in behavioral strategy control

The selective engagement of cholinergic and noradrenergic systems by VNS has important implications for adaptive behavior. Through its concurrent effects on these neuromodulatory systems, VNS can dynamically adjust the balance between feedforward sensory processing and feedback predictions based on contextual demands. In novel environments, VNS may optimize learning through two complementary mechanisms: cholinergic enhancement of feedforward prediction errors promotes effective model updating, whereas noradrenergic modulation facilitates adaptive model revision through adjusted state transition precision (Shine, 2019; Munn et al., 2021; Shine et al., 2021). This dual modulation benefits situations that require new learning or adaptation. However, in stable environments where established internal models guide behavior, VNS-induced enhancement of feedforward processing might unnecessarily increase the sensitivity to prediction errors, potentially disrupting performance.

These modulatory effects also influence the exploration-exploitation trade-off in behavior; exploitation facilitates focused task performance, while exploration promotes sampling of alternative behaviors (Aston-Jones and Cohen, 2005). The noradrenergic system operates in two distinct modes that regulate behavioral strategies (Aston-Jones and Cohen, 2005). In the phasic mode, neurons exhibit transient activation patterns time-locked to task-relevant decision processes, which promote exploitation by facilitating focused attention and optimal task performance when reward utility is high. In contrast, the tonic mode is characterized by a sustained elevation of baseline activity, which emerges when reward utility diminishes. This elevated tonic activity enhances responsiveness to task-irrelevant stimuli, thereby promoting the exploration of alternative behavioral opportunities. These distinct firing patterns may differentially engage in neural processing within the cortical hierarchy. Moderate tonic LC activation preferentially engages higher-order associative regions, whereas burst-like stimulation enhances the activity in lower-order sensory regions (Grimm et al., 2024). These differential effects on cortical processing may contribute to flexible adjustments in behavioral strategies, although precise mapping between experimental stimulation patterns and naturally occurring LC activity patterns requires further investigation. Indeed, VNS parameters can differentially modulate LC firing modes. While standard VNS (10–30 Hz) induces consistent activation of specific LC neurons, bursting VNS (300–350 Hz) promotes synchronous firing between LC neurons, although detailed analysis of temporal firing patterns in individual LC neurons remains to be elucidated (Farrand et al., 2023).

The cholinergic system enables efficient task performance by modulating sensory gain in task-relevant neural circuits through precise spatiotemporal control over cortical information processing. In the basal forebrain, two distinct populations of cholinergic neurons differentially contribute to behavioral control through different firing patterns. Burst-firing cholinergic neurons generate precisely timed responses to behaviorally salient events and can synchronously activate cortical circuits, while regular-firing neurons show distinct temporal activity patterns that correlate with successful performance in sensory detection tasks (Laszlovszky et al., 2020). Recent studies have shown that VNS may engage these cholinergic mechanisms by robustly modulating basal forebrain activity with precise temporal dynamics (Bowles et al., 2022; Martin et al., 2024). Specifically, VNS-induced activation of basal forebrain neurons begins during stimulation and can persist for several seconds, leading to the activation of cortically projecting cholinergic axons and subsequent behavioral improvements (Martin et al., 2024).

Based on these distinct neuromodulatory mechanisms, VNS may dynamically regulate behavioral strategies through parallel engagement of both systems: noradrenergic control determines behavioral state transitions, while cholinergic modulation enhances task-specific neural processing. These dual mechanisms can be conceptualized within the energy landscapes of cortical activity, a probabilistic framework representing the stability of brain states, with noradrenergic effects flattening the landscape to enable flexible transitions, whereas cholinergic modulation deepens specific valleys to stabilize newly established brain states (Munn et al., 2021). Through this dual neuromodulatory influence, VNS likely enhances adaptive behavior by simultaneously promoting both exploration of novel environmental contexts and exploitation of task-relevant sensory information. This mechanistic framework suggests that optimal VNS application requires careful consideration of both the task demands and environmental stability. Although VNS might enhance learning and adaptation in novel environments by strengthening feedforward signaling of prediction errors, its application during periods requiring stable performance could be counterproductive by disrupting established predictive models.

## Cognitive implications of VNS-induced circuit modulation

In a recent meta-analysis, invasive VNS produced limited cognitive benefits in patients with epilepsy, showing no significant improvements in overall cognitive performance, executive function, attention, and memory (Kong et al., 2024). Similarly, transcutaneous auricular VNS (taVNS) demonstrated only a small effect size (Hedges’ *g* = 0.21, 95% CI = 0.12–0.29) on overall cognitive performance in healthy individuals (Ridgewell et al., 2021). These heterogeneous effects of VNS on cognitive function might be understood through differential modulation of feedforward and feedback processing. While VNS consistently enhances feedforward signals through cholinergic activation, this enhancement does not uniformly translate to improved cognitive performance, possibly due to the fundamental importance of balanced feedforward and feedback signals in cognitive processing (Shine et al., 2021; Halvagal and Zenke, 2023).

The cognitive impact of VNS appears to be highly dependent on task context. In well-practiced tasks such as recalling memorized information or performing familiar motor skills, the brain predominantly utilizes established internal models through feedback processing. Under these conditions, VNS-enhanced feedforward signaling may introduce unnecessary interference by increasing the sensitivity to prediction errors. This interference has been observed in verbal memory tasks and cognitive flexibility assessments that rely on established internal representations (Ghacibeh et al., 2006; Mertens et al., 2020). Conversely, VNS may enhance performance during new skill acquisition or environmental adaptation such as learning a new language or mastering novel motor patterns, as demonstrated in a study in which taVNS significantly improved adults’ ability to learn novel letter-sound correspondences in unfamiliar orthographies (Thakkar et al., 2020). Vagus nerve stimulation enhances reinforcement learning, particularly in individuals with lower extraversion traits (Weber et al., 2021). The mechanism may involve modulation of the balance from internally generated representations toward externally driven sensory inputs, i.e., from prediction to prediction error.

The variable effects of VNS on cognitive function may stem not only from the task context but also from the stimulation parameters used across studies (Vonck et al., 2014). Cognitive benefits may be achieved at lower current intensities than those typically employed in epilepsy and depression (Broncel et al., 2020). Studies using implanted VNS systems have demonstrated that moderate stimulation intensities around 0.4–0.5 mA have been shown to enhance recognition memory, while higher intensities (0.75–2.5 mA) either fail to improve or may actually impair memory function (Clark et al., 1999; Helmstaedter et al., 2001). This inverted U-shaped response pattern was observed in VNS-induced hippocampal plasticity. Vagus nerve stimulation optimally facilitates long-term potentiation (LTP), a form of synaptic plasticity underlying learning and memory, in the dentate gyrus of rats at 0.4 mA, while both lower (0.2 mA) and higher (0.8 mA) stimulation intensities produce weaker effects (Zuo et al., 2007). This intensity-dependent pattern appears to be mediated by differential levels of noradrenaline release, suggesting that VNS parameters effective for seizure control may inhibit feedback processing through excessive noradrenergic modulation.

The temporal precision of the VNS application has emerged as a critical factor in determining its cognitive effects. Unlike animal studies, many human studies have applied VNS without pairing with a specific task-relevant stimulus (Kong et al., 2024). Vagus nerve stimulation efficacy may be optimized when precisely synchronized with specific cognitive operations (Bowles et al., 2022; Martin et al., 2024). This precise timing is crucial for optimal acetylcholine release, which is necessary to enhance task-relevant feedforward signaling that updates the internal model. Vagus nerve stimulation, as well as stimulation of the cholinergic nucleus basalis, paired with a specific frequency tone, induces cortical map expansion in a region corresponding to the paired tone frequency in the auditory cortex (Kilgard and Merzenich, 1998a; Engineer et al., 2011), which improves perceptual learning but is not necessary for improved tone discrimination (Reed et al., 2011). A similar cortical map expansion is observed at the early stage of learning in auditory operant conditioning (Takahashi et al., 2010; Takahashi et al., 2011), and this map expansion is associated with the diversity of tone-evoked neural responses (Takahashi et al., 2013). Early learning primarily relies on bottom-up sensory pathways (Makino and Komiyama, 2015); therefore, it may benefit from acetylcholine-mediated enhancement induced by VNS (Bowles et al., 2022; Martin et al., 2024). Late-phase learning, which requires the formation of novel neural activity patterns through feedback processing, may not benefit from VNS enhancement of feedforward circuits (Carroll et al., 2024). Thus, the temporal precision of VNS with respect to task-relevant stimuli may be critical at the early stage of learning.

In contrast to timing-dependent effects, VNS may also fundamentally enhance perceptual precision through the modulation of arousal systems, independent of specific task demands (Collins et al., 2021). Both the noradrenergic and cholinergic systems activated by VNS induce widespread cortical excitation and strongly influence the arousal state. Heightened arousal levels enhance both single-neuron and population-level sensory encoding, characterized by increased signal-to-noise ratios and reduced noise correlations, while altering cortical activity patterns by reducing low-frequency oscillations and enhancing gamma-band synchronization (Vinck et al., 2015). Different levels of arousal also trigger distinct patterns of cholinergic modulation. During moderate arousal, acetylcholine release becomes more coordinated across cortical regions, whereas high-arousal states lead to profound decorrelation of cholinergic signals (Lohani et al., 2022). Such state-dependent changes in cholinergic signaling may optimize cognitive processing by dynamically adjusting the cortical network coordination across various behavioral states.

The insights from these diverse studies on VNS and cognition can be integrated within our feedforward-feedback modulation framework. Through this lens, we can explain why VNS produces variable cognitive effects: tasks requiring novel sensory processing and early learning benefit from VNS because it enhances feedforward prediction error signals via cholinergic activation, facilitating the updating of internal models. Conversely, well-practiced tasks that rely heavily on established internal models may show limited improvement or even impairment with VNS since the enhanced feedforward signaling introduces disruptive prediction errors into otherwise efficient feedback-dominated processing. Optimizing VNS for cognitive enhancement requires careful consideration of multiple factors, including cognitive context, stimulation parameters (with moderate intensities likely providing optimal balance between cholinergic and noradrenergic effects on feedforward-feedback circuits), and temporal dynamics (precise timing may selectively enhance feedforward processing during critical periods of prediction error generation). Future applications in behavioral research should focus on precisely targeting VNS to specific cognitive phases where enhanced feedforward processing would most effectively update internal models (e.g., during initial exposure to novel stimuli in perceptual learning tasks), while avoiding cognitive phases dominated by feedback processing (e.g., during retrieval of well-established memories or complex problem-solving using existing knowledge). This mechanistic approach may help to resolve the current heterogeneity in cognitive outcomes and lead to more effective therapeutic applications of VNS.

## Clinical implications of VNS-induced circuit modulation

The enhancement of feedforward signaling by VNS may facilitate the update of internal models and provide a mechanistic framework for understanding its diverse therapeutic applications (Figure 2B). While this feedforward enhancement may be particularly relevant for conditions requiring internal model updating such as depression and stroke rehabilitation, VNS could also modulate aberrant feedback signaling that contributes to seizure propagation in epilepsy. This balanced modulation of the feedforward and feedback pathways helps restore optimal circuit dynamics across different neurological and psychiatric conditions.

### Epilepsy

Vagus nerve stimulation treatment outcomes in patients with epilepsy remain notably heterogeneous, with the responder rate (≥50% reduction in seizure frequency) reaching approximately 60% within 2–3 years of therapy (Kawai et al., 2017). Although VNS has been in clinical use for several decades, reliable predictive markers of therapeutic response remain elusive (Workewych et al., 2020; Clifford et al., 2024). Recent evidence suggests that alterations in thalamocortical connectivity may play a crucial role in determining the treatment outcomes. A diffusion tensor imaging (DTI) study in 56 children showed that VNS responders demonstrated greater preoperative integrity of thalamocortical pathways than non-responders (Mithani et al., 2019). A functional magnetic resonance imaging (fMRI) study in 21 medically intractable pediatric epilepsy patients reported that pre-surgically enhanced thalamocortical connectivity has also been associated with favorable VNS responses (Ibrahim et al., 2017). Indeed, an early PET imaging study demonstrated that increased thalamic blood flow during acute VNS was strongly correlated with improved seizure outcomes, supporting the thalamus as a key therapeutic target (Henry et al., 1999). Through our feedforward-feedback modulation framework, these findings suggest that VNS may exert its anti-seizure effects by enhancing the structural and functional connectivity of thalamocortical feedforward pathways. Further studies are needed to elucidate how VNS specifically alters information flow between the thalamus and cortex in patients with epilepsy.

Beyond thalamocortical circuits, recent laminar recordings during human seizures have revealed distinct patterns of cortical layer engagement during seizure initiation and propagation (Bourdillon et al., 2024). In the seizure onset zone, epileptic activity originates and persists in the infragranular layers, suggesting that the pathological activity patterns in these deep layers may drive seizure initiation. In areas outside the seizure onset zone, seizure activity predominantly engages layer I before descending to deeper layers, which corresponds to the anatomical pathway of the cortical feedback signals. Within our feedforward-feedback modulation framework, VNS may exert its therapeutic effect by enhancing activity in the supragranular layers, which primarily process feedforward signals, thereby counterbalancing the pathological feedback-dominated activity in the infragranular layers. The delayed therapeutic effect often observed with VNS might reflect the time required for the gradual rebalancing of these laminar-specific circuit dynamics through chronic stimulation.

### Depression

Major depressive disorder (MDD) is a complex disorder involving multiple pathophysiological alterations, including changes in brain structure and function, inflammation, gut-brain axis, and hypothalamic–pituitary–adrenal axis, with at least 30% of patients showing an inadequate response to multiple medications (Marx et al., 2023; McIntyre et al., 2023). While the monoamine hypothesis has historically dominated our understanding of MDD focusing on serotonin and noradrenaline systems, this framework cannot fully account for the delay between pharmacological action and clinical improvement, or the heterogeneous treatment responses. Although VNS exerts anxiolytic and antidepressant-like behavioral effects through serotonergic and noradrenergic pathways in rats (Furmaga et al., 2011), the underlying mechanism of action in MDD remains unclear (Nemeroff et al., 2006; Grimonprez et al., 2015; Carreno and Frazer, 2017).

Functional MRI studies have demonstrated that MDD involves disrupted network regulation across multiple brain regions, rather than dysfunction in any single neurotransmitter system. Major depressive disorder is associated with hyperconnectivity within the default mode network, which is involved in self-referential processes and maladaptive depressive rumination (Hamilton et al., 2011; Kaiser et al., 2015; Hamilton et al., 2015; Kawakami et al., 2024). Moreover, MDD exhibits a distinctive pattern of network imbalance: decreased connectivity between the frontoparietal network (involved in cognitive control) and dorsal attention network (involved in external attention), along with increased connectivity between the frontoparietal network and default mode network (involved in internal thought processes) (Kaiser et al., 2015). This suggests that the core features of depression may not simply arise from increased or decreased activity in specific regions but rather from an imbalance in network interactions. Such network dysregulation may help explain two characteristic features of depression: an excessive focus on internal thoughts and reduced engagement with the external environment. Furthermore, a resting-state fMRI study revealed that patients with MDD show reduced functional connectivity within both the visual and auditory networks, as well as between these networks, indicating altered sensory processing (Lu et al., 2020).

From our feedforward-feedback modulation framework, this network dysregulation in MDD can be conceptualized as an imbalance favoring feedback processing over feedforward information flow. The hyperconnectivity within the default mode network may represent excessive top-down feedback processing that drives depressive rumination, while the reduced connectivity in sensory networks may reflect diminished bottom-up feedforward processing of external stimuli. Based on these network-level disruptions in MDD, VNS may exert its therapeutic effects by restoring this imbalanced neural circuitry through differential modulation of feedforward and feedback pathways. Specifically, by enhancing feedforward information flow through neural circuits, VNS could help shift attention from internal rumination toward external environmental inputs. Indeed, research has demonstrated that taVNS can significantly modulate default mode network connectivity in patients with MDD (Fang et al., 2016), suggesting that VNS can be an effective intervention for rebalancing maladaptive neural circuitry in MDD.

### Stroke rehabilitation

Beyond its application in psychiatric disorders, VNS has emerged as a promising therapeutic approach in neurological rehabilitation. Clinical trials have demonstrated the efficacy of VNS paired with rehabilitation for improving upper limb function in patients with upper limb impairment at least 4 months after ischemic stroke (Dawson et al., 2021; Kimberley et al., 2018). A pivotal randomized, triple-blind trial showed that VNS paired with rehabilitation significantly improved arm function compared to rehabilitation with sham stimulation, with 47% of VNS-treated patients achieving clinically meaningful responses in the Fugl-Meyer Assessment-Upper Extremity (FMA-UE) score versus 24% in the control group (Dawson et al., 2021). The therapeutic protocol involves precise timing of VNS lasting 0.5 s with specific rehabilitation movements including reach and grasp exercises. This improvement in chronic stroke patients, with a mean time since stroke of approximately 3 years, is particularly noteworthy because functional improvement at this chronic stage is unexpected and this time point corresponds to the onset of the greatest functional decline in stroke survivors (Dhamoon et al., 2009). Recent long-term follow-up data also demonstrate that VNS-induced improvements not only persist, but continue to increase over the years (Francisco et al., 2023).

Animal studies have elucidated the neurobiological mechanisms underlying these clinical benefits. Vagus nerve stimulation drives task-specific plasticity in the motor cortex with paired VNS movement training, leading to a significant expansion of movement representations in motor maps (Porter et al., 2012). In a rat model of stroke, VNS enhances synaptic connectivity in motor networks and doubles motor recovery compared with rehabilitation alone (Meyers et al., 2018). This VNS-dependent plasticity appears to involve cholinergic signaling, as lesions of the nucleus basalis, the primary source of cortical acetylcholine, prevent VNS-dependent enhancement of motor cortex plasticity, and cortical cholinergic depletion blocks VNS-driven motor and sensory recovery (Hulsey et al., 2016; Meyers et al., 2019). Furthermore, VNS promotes skilled motor learning via cholinergic reinforcement, mediated by selective modulation of outcome-relevant neural circuits in the motor cortex (Bowles et al., 2022). Its therapeutic benefit is optimized when stimulation is precisely timed with successful movements (Khodaparast et al., 2014).

These findings can be mechanistically explained through our feedforward-feedback modulation framework. VNS appears to enhance sensorimotor feedforward signaling primarily through cholinergic modulation. When precisely timed with rehabilitation exercises, VNS-driven cholinergic reinforcement strengthens outcome-relevant neural circuits in the motor cortex. This selective enhancement of feedforward processing enables more efficient updating of internal models, particularly those related to motor representations in the stroke-affected brain. This rebalancing of feedforward-feedback dynamics may be particularly effective in chronic stroke, in which maladaptive feedback processing could interfere with motor learning and functional recovery.

In stroke rehabilitation, VNS is likely to support the reorganization of the somatopic map in the sensorimotor cortex. Such cortical reorganization may be a common mechanism for the treatment of other pathological conditions in the cortex. For example, deafferentation due to hearing loss disrupts the tonotopic map in the auditory cortex, resulting in tinnitus (Mühlnickel et al., 1998; Eggermont, 2015; Wake et al., 2024), and similarly the disorganization of somatopic maps in the somatosensory cortex is associated with phantom pain following limb amputation (Flor et al., 1995). Vagus nerve stimulation-assisted reorganization of these pathological cortices may be a potential treatment in the future (Engineer et al., 2011; De Ridder et al., 2021).

## Methodological considerations in evaluating VNS interventions: comparing icVNS and taVNS

The clinical evidence discussed above for epilepsy, depression, and stroke rehabilitation has primarily been established using invasive cervical VNS (icVNS). When evaluating VNS interventions for these diseases, it is crucial to distinguish between icVNS and non-invasive taVNS. While icVNS directly stimulates vagal nerve fibers, taVNS delivers stimulation through the skin of the external ear, potentially affecting multiple neural pathways beyond the vagus nerve (Mahadi et al., 2019). This anatomical and mechanistic distinction has important implications for the therapeutic efficacy. A recent commentary has highlighted that combining these distinct interventions in meta-analyses may lead to misleading conclusions regarding their relative effectiveness, emphasizing the need for intervention-specific evaluation of clinical outcomes (Malakouti et al., 2024). Both interventions activate the nucleus tractus solitarius (NTS), which receives the majority of vagal afferents but with notably different patterns across its subdivisions. The dorsolateral regions show stronger activation with icVNS, whereas the dorsomedial regions respond more robustly to taVNS (Ali et al., 2024). Detailed electrophysiological investigations have further demonstrated that, while taVNS and icVNS produce comparable overall NTS activation patterns, their effects at the single-neuron level can be markedly different and sometimes opposite (Owens et al., 2024). Of particular interest is the finding that taVNS produces more pronounced activation of the spinal trigeminal nucleus (Sp5), a structure known to receive direct projections from the auricular branch of the vagus nerve, than NTS activation. These observations provide strong evidence that these interventions may engage different neuronal pathways to achieve therapeutic effects. Intriguingly, transcutaneous stimulation of the cervical region has been shown to deactivate Sp5 (Frangos and Komisaruk, 2017), suggesting potentially different mechanisms of action between the cervical and auricular approaches. This modulatory pattern may help explain the therapeutic benefits of non-invasive cervical VNS in acute migraine attacks (Goadsby et al., 2017; Brennan and Pietrobon, 2018; Ashina, 2020; Puledda et al., 2023), although the precise mechanisms remain to be fully elucidated.

These findings underscore a critical point: while icVNS and taVNS may appear to activate similar brain regions at the macro level such as fMRI (Badran et al., 2018), their effects on specific brainstem nuclei follow distinct patterns. This nuclei-specific targeting indicates the need for careful consideration when interpreting the therapeutic mechanisms of non-invasive VNS approaches, and highlights that we should not assume complete mechanistic equivalence with invasive VNS, even though they partially share some clinical benefits. Future studies are needed to systematically investigate whether our proposed feedforward-feedback modulation framework, primarily derived from icVNS animal models, applies equally to taVNS.

## Conclusion

The hypothesis of the VNS-induced differential modulation of neural connections offers a novel perspective on the mechanisms underlying this neuromodulatory intervention. By selectively amplifying the feedforward signaling of prediction errors while weakening the influence of feedback circuits, VNS might facilitate the brain’s ability to minimize prediction errors and optimize its internal model. This mechanism is consistent with the organization of neuromodulatory systems in the cerebral cortex, particularly cholinergic and noradrenergic projections, which are predominantly affected by VNS. This integrative view provides a potential explanation for the diverse effects of VNS and opportunities for computational research on neuromodulation. Future investigations focusing on the layer-specific actions of VNS, its interactions with neuromodulatory systems, and its impact on predictive brain processes may lead to more targeted and effective therapies. Taken together, this perspective underscores the potential of VNS as both a therapeutic tool and an approach for investigating the fundamental principles of neural information processing.

## Data Availability

The original contributions presented in the study are included in the article/supplementary material, further inquiries can be directed to the corresponding author.
